# The value of preoperative systemic immune-inflammation index in predicting vascular invasion of hepatocellular carcinoma: a meta-analysis

**DOI:** 10.1590/1414-431X202010273

**Published:** 2021-02-26

**Authors:** YiFeng Wu, ChaoYong Tu, ChuXiao Shao

**Affiliations:** 1 Department of Hepatobiliary and Pancreatic Surgery, The First Affiliated Hospital of Wenzhou Medical University, Wenzhou, Zhejiang Province, China; 2 Department of Hepatobiliary and Pancreatic Surgery, The Fifth Affiliated Hospital of Wenzhou Medical University, Lishui Municipal Central Hospital, Lishui, Zhejiang Province, China

**Keywords:** Hepatocellular carcinoma, Meta-analysis, Vascular invasion, Microvascular invasion, Systemic immune-inflammation index

## Abstract

Vascular invasion and systemic immune-inflammation index (SII) are risk factors for the prognosis of patients with hepatocellular carcinoma. At present, the correlation between the two is not clear. This meta-analysis explored the relationship between preoperative SII and vascular invasion in patients with hepatocellular carcinoma. According to the search formula, the Pubmed, Embase, Cochrane, Web of Science, and CNKI databases were searched for the relevant research until March 2020. After the quality evaluation of the included literature, the odds ratio (OR) and its corresponding 95% confidence interval (CI) were used as the effect measure. Stata 15. 0 software was used for statistical analysis. The meta-analysis eventually included seven retrospective cohort studies of 3583 patients with hepatocellular carcinoma. The results showed that the choice of SII cut-off value affects SII's efficiency in predicting the risk of vascular invasion. In the cohort of studies with appropriate SII cut-off value, the high SII preoperative group had a higher risk of vascular invasion (OR=2.62; 95%CI: 2.07-3.32; P=0.000) and microvascular invasion (OR=1.82; 95%CI: 1.01-3.25; P=0.045) than the low SII group. The tumor diameter (OR=2.88; 95%CI: 1.73-4. 80; P=0.000) of the high SII group was larger than that of the low SII group. There was no publication bias in this study (Begg's test, P=0.368). As a routine, cheap, and easily available index, SII can provide a certain reference value for clinicians to evaluate vascular invasion before operation.

## Introduction

With a concealed onset and high incidence, primary liver cancer is the sixth most common malignant tumor globally. The main pathological type of primary liver cancer is hepatocellular carcinoma (HCC) (1). At present, HCC's multidisciplinary treatment is advocated, including hepatectomy, liver transplantation, transcatheter arterial chemoembolization, local ablation, and targeted therapy. However, even during hepatectomy, about 70% of patients with HCC have recurrence and metastasis 5 years after the operation (2). Vascular invasion is a significant risk factor of high postoperative recurrence and low long-term survival rate in patients with HCC (3). Therefore, the early prediction of vascular invasion before the operation is of great value to guide the choice of intraoperative factors, the determination of resection margin, combination of adjuvant therapy, and the screening of candidates for liver transplantation. Vascular invasion includes macrovascular invasion and microvascular invasion (MVI). At present, with the help of imaging and serological and pathological examination, the macrovascular invasion of patients with hepatocellular carcinoma can be evaluated before the operation. However, due to technical limitations, the diagnosis of MVI can only rely on postoperative histopathology.

As one of the ten characteristics of tumors (4), tumor-related inflammation has become a hot field of oncology research. Since systemic immune-inflammation index (SII) was first proposed by Hu et al. (5) in 2014, it has been proven to be closely related to the prognosis of patients with HCC in a variety of treatment measures, including surgical resection (5), transarterial chemoembolization (6), liver transplantation (7), and sorafenib administration (8). However, the relationship between SII and vascular invasion is not clear, and there are even some contradictory conclusions. Therefore, we conducted a meta-analysis of published studies to further explore the relationship between preoperative SII levels and vascular invasion. Furthermore, we also analyzed the correlation between preoperative SII and tumor diameter.

## Material and Methods

### Search strategy

Electronic retrieval was used. According to the pre-determined retrieval formula, two authors searched the Pubmed, Embase, Cochrane, Web of Science, and CNKI databases. The search date was up to March 2020. According to different databases' characteristics, the subject words were combined with free words and keywords for comprehensive retrieval. The keywords searched were “liver cancer” or “hepatocellular carcinoma” or “HCC” and “systemic immune inflammation index” or “SII” or “neutrophils × platelets/lymphocytes”. The search strategy is shown in Supplementary Table S1.

### Inclusion and exclusion criteria of literature

Inclusion criteria: 1) the reference reported the relationship between pre-treatment (non-operative and operative) SII and vascular invasion in patients with HCC; 2) all patients were diagnosed as HCC by liver biopsy or histopathology; 3) the patients were divided into low SII group and high SII group according to the cutoff-value; and 4) the reference provided preoperative SII grouping and vascular invasion data.

Exclusion criteria: 1) patients with non-hepatocellular carcinoma diagnosed by histopathology; 2) reference was a summary, case report, comment, and meeting summary, etc.; 3) the full text of the reference could not be obtained or the reference quality could not be evaluated; and 4) reference with duplicate data from a database (only the study with the largest number of patients was included).

### Data extraction and quality evaluation

According to the pre-designed scheme, all references were independently screened, and data were extracted and evaluated by two authors (Wu and Tu). Any inconsistencies were resolved through discussion or by a third party. Data extracted from the reference included author, date of publication, country of the study population, sex ratio, age, total number of studies, cut-off value of SII, vascular invasion, and tumor diameter in high and low SII groups. The Newcastle-Ottawa scale (NOS) was used for quality evaluation. The scale has three evaluation parameters and eight items, including study population selection, inter-group comparability, and outcome measurement. The total score is nine and a score ≥6 was regarded as high-quality reference.

### Statistical analysis

Statistical analysis was performed by Stata 15.0 software (StataCorp, USA). The data in this study are reported as odds ratio (OR) and 95%CI. The heterogeneity test of the included studies was conducted, and the I^2^ and P value were used for evaluation. If there was no evidence of significant heterogeneity (I^2^<50% and P>0.1), the fixed-effect model was used. Otherwise, the random effect model was used. To evaluate the results' stability, one study was omitted at a time in the sensitivity analysis. Begg's test was used to assess publication bias. If P<0.05, there was significant publication bias.

## Results

### Research selection process and quality evaluation

**Figure 1:**
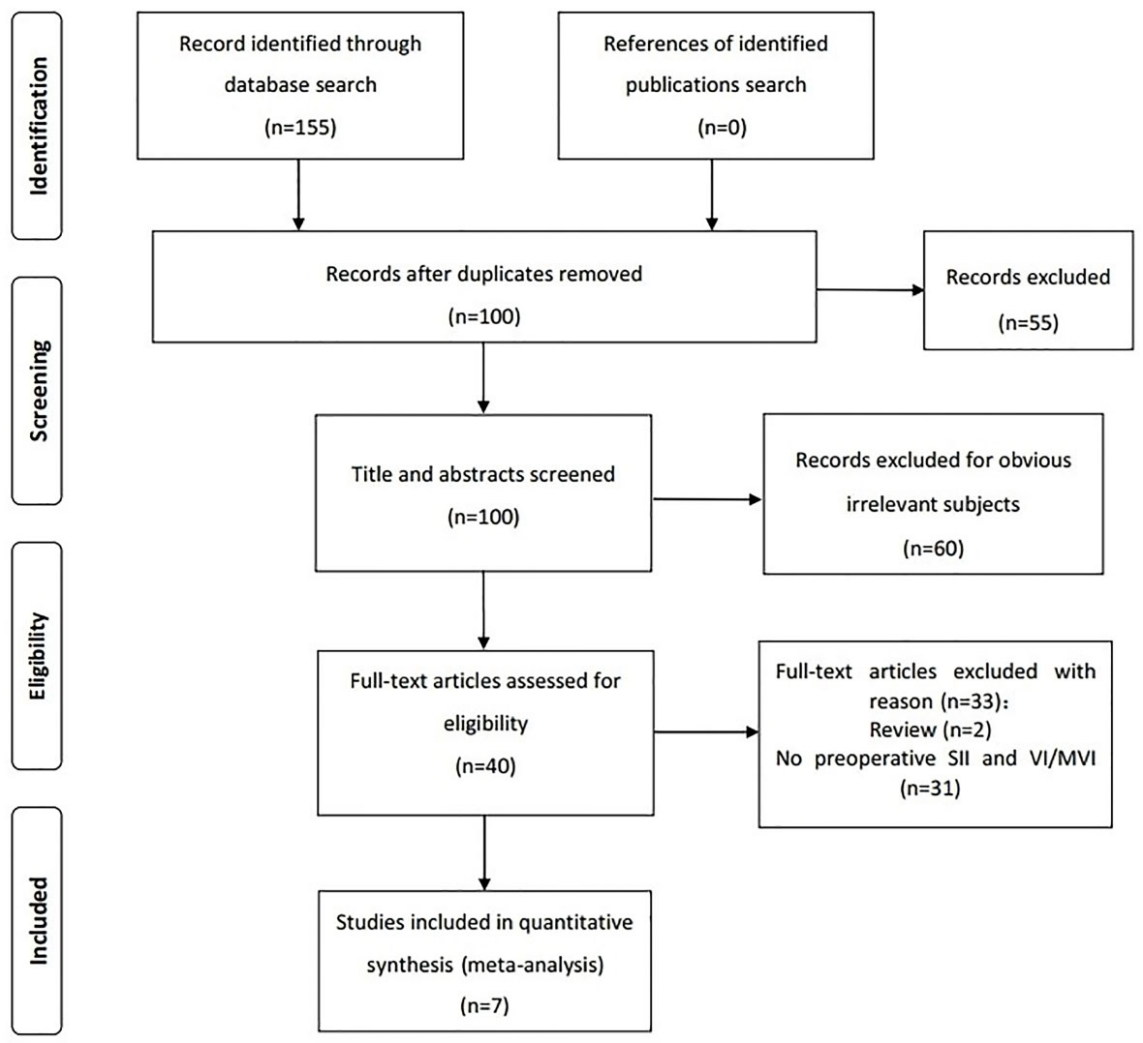
Literature screening process and results.

The initial search produced 155 studies based on the search strategy, from which 55 duplicates and 60 studies whose research purposes were not consistent with this meta-analysis were excluded. The full text of the remaining 40 references was carefully read and 33 references were excluded for the following reasons: data on preoperative SII and vascular invasion were not reported (n=31) and articles were reviews (n=2). Finally, seven studies (5,7,9–13) met the inclusion criteria, all of which were published between 2014 and March 2020 (Figure 1). The quality scores of the studies assessed by the NOS ranged from 6 to 8 (average 7 points) (Table 1).

**Table 1:** Newcastle-Ottawa scale quality score of each study.

First author	Publication year	Study design	Selection	Comparison	Exposure/Outcome	Total quality score
Chan et al. (9)	2019	RCS	⋆⋆⋆	⋆	⋆⋆⋆	7
Fu et al. (7)	2018	RCS	⋆⋆⋆	⋆	⋆⋆⋆	7
Hu et al. (5)	2014	RCS	⋆⋆⋆	⋆	⋆⋆⋆	7
Huang et al. (10)	2019	RCS	⋆⋆⋆	⋆	⋆⋆	6
Pang et al. (11)	2018	RCS	⋆⋆⋆	⋆⋆	⋆⋆⋆	8
Wang et al. (12)	2019	RCS	⋆⋆⋆	⋆	⋆⋆⋆	7
Pan et al. (13)	2018	RCS	⋆⋆⋆	⋆⋆	⋆⋆	7

The Newcastle-Ottawa scale has three evaluation parameters and eight items, including study population selection, inter-group comparability, and outcome measurement. RCS: retrospective cohort study.

### Description of the studies

The seven studies included in this meta-analysis were retrospective cohort studies involving data from 3583 patients with HCC (Supplementary Table S2). One was from Hong Kong (9), one was from Taiwan (10), and the other five were from China (5,7,11–13). Three studies (5,9,11) reported the relationship between preoperative SII level and vascular invasion, four studies (7,10,12,13) reported the relationship between preoperative SII and MVI, and four studies (5,7,11,13) reported the relationship between preoperative SII and tumor diameter (5 cm was the cut-off value).

### Relationship between preoperative SII and vascular invasion

**Figure 2:**
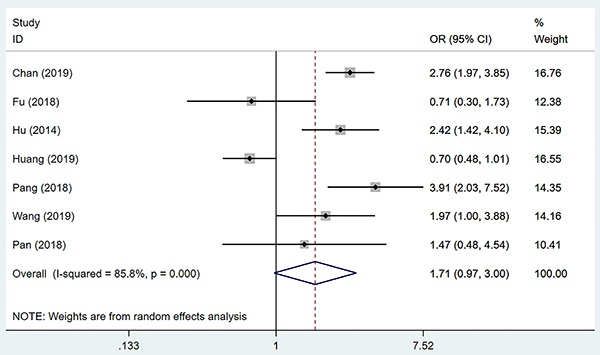
Forest plot of the relationship between preoperative systemic immune-inflammation index and vascular invasion in patients with hepatocellular carcinoma. See Supplementary Table S2 for reference numbers.

Seven studies (5,7,9–13) reported the relationship between SII and vascular invasion (including MVI). Considering that there was significant heterogeneity (I^2^: 85.8%, P=0.000), the random effect model was adopted. The combined OR showed that there was no significant difference in the risk of vascular invasion between high SII group and low SII group in HCC patients (OR=1.71, 95%CI: 0.97-3.00, P=0.062) (Figure 2).

**Figure 3:**
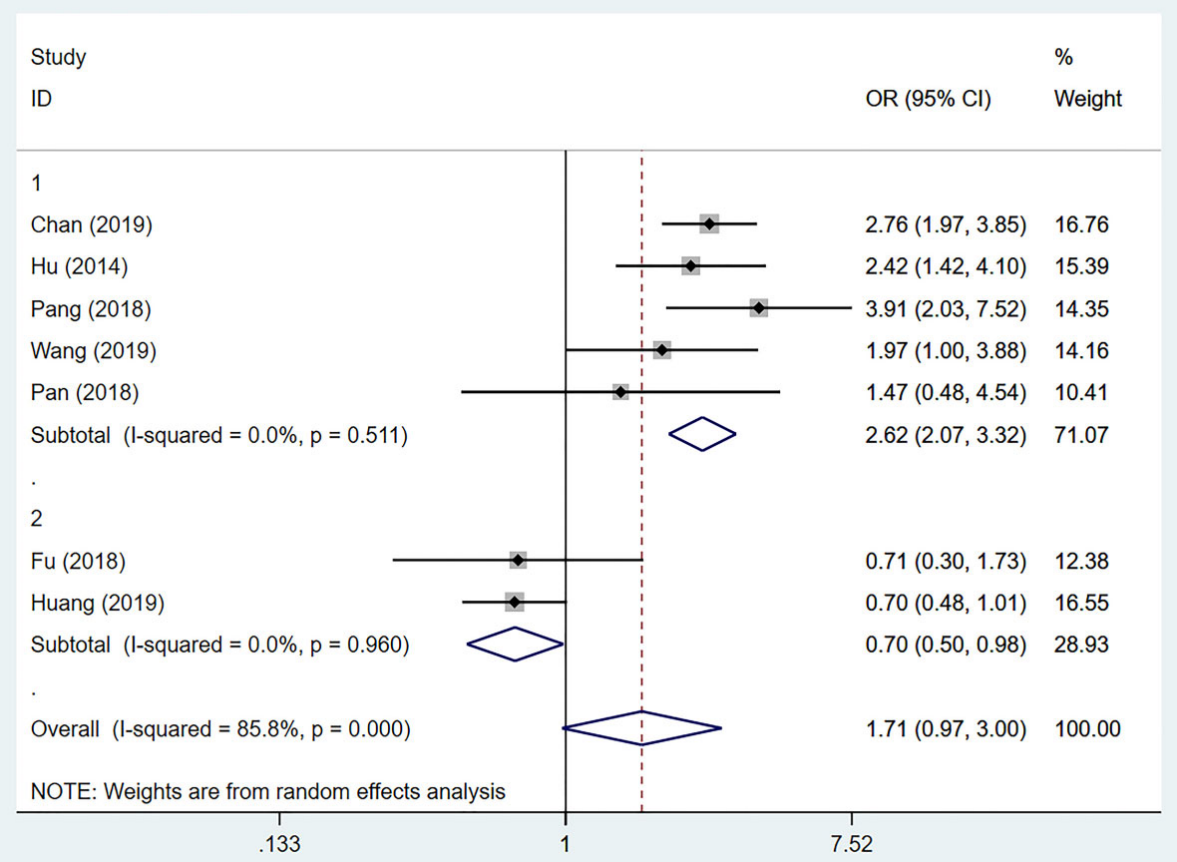
Subgroup analysis of the relationship between preoperative systemic immune-inflammation index (SII) and vascular invasion according to the preoperative SII cut-off value. See Supplementary Table S2 for reference numbers.

Because of the considerable heterogeneity, the study used subgroup analysis to further investigate the source of heterogeneity. After carefully reading the full text, it was found that the cut-off values of SII in Fu et al. (7), Huang et al. (10), Chan et al. (9), Hu et al. (5), Pang et al. (11), Wang et al. (12) and Pan et al. (13) were 226, 160, 330, 330, 340.66, 305, and 360.9 respectively. The cut-off values of SII in Fu et al. (7) and Huang et al. (10) were significantly lower than that in the other five studies. Therefore, according to SII's cut-off value, Fu et al. (7) and Huang et al. (10) were placed into the low cut-off value group, and the other five studies were placed into the high cut-off value group. The data collected by Fu et al. (7) and Huang et al. (10) showed that the risk of vascular invasion in the high SII group was not higher than that in the low SII group (OR=0.70, 95%CI: 0.50-0.98, P=0.040), and there was no significant heterogeneity (I^2^=0.0%, P=0.960). The summary data of the remaining five studies showed that the high SII group was more likely to have vascular invasion than the low SII group in HCC patients (OR=2.62, 95%CI: 2.07-3.32), and there was no significant heterogeneity (I^2^=0.0%,P=0.511) (Figure 3).

**Figure 4:**
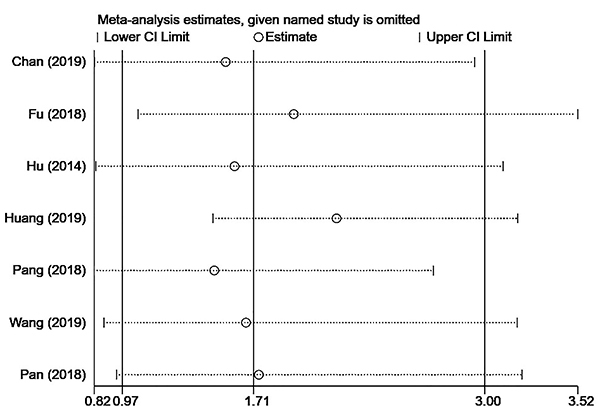
Sensitivity analysis of the relationship between preoperative systemic immune-inflammation index and vascular invasion. See Supplementary Table S2 for reference numbers.

This study also carried out a subgroup analysis according to the endpoint vascular invasion or MVI. The results showed that among those with or without vascular invasion, the high SII group was more likely to have vascular invasion than the low SII group (OR=2.82, 95%CI: 2.18-3.66), and there was no significant heterogeneity (I^2^=0.0%, P=0.523). In those with or without microvascular invasion, the high SII group was not more prone to microvascular invasion than the low SII group (OR=1.04,95%CI: 0.59-1.86, P=0.884) and there was significant heterogeneity (I^2^=85.8%, P=0.000) (Table 2). The results showed that the endpoint was not the cause of the heterogeneity. Finally, the sensitivity analysis of this study (Figure 4), showed that the studies of Fu et al. (7) and Huang et al. (10) were the sources of heterogeneity. Combined with the sensitivity analysis results and subgroup analysis of SII cut-off value, it was suggested that the SII cut-off value might be the primary source of heterogeneity in this study.

**Table 2:** Results of subgroup analysis for preoperative systemic immune-inflammation index based on the cut-off value and the endpoint [vascular invasion (VI) and micro-vascular invasion (MVI)].

Factor	Included studies	HR 95%CI	P value	I^2^ (%)	P value for heterogeneity
Cut-off value					
High cut-off value	5	2.62 (2.62-3.32)	0.00	0.0	0.511
Low cut-off value	2	0.70 (0.50-0.98)	0.04	0.0	0.96
Endpoint					
VI	3	2.82 (2.18-3.66)	0.00	0.0	0.523
MVI	4	1.04 (0.59-1. 6)	0.883	62.5	0.046

VI, vascular invasion; MVI, micro-vascular invasion.

### Relationship between preoperative SII and microvascular invasion

**Figure 5:**
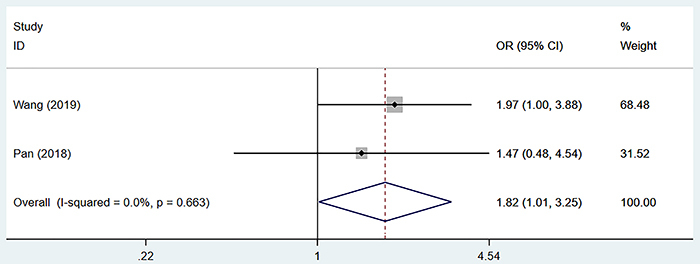
Forest plot of the relationship between preoperative systemic immune-inflammation index and microvascular invasion. See Supplementary Table S2 for reference numbers.

Excluding the two studies that caused heterogeneity (7,10) from the remaining five studies (5,9,11–13), two studies (12,13) reported the relationship between SII and MVI. Since no apparent heterogeneity was observed (I^2^=0.0%, P=0.663), a fixed-effect model was used. The summary analysis showed that the high SII group was more prone to MVI (OR=1.82, 95%CI: 1.01-3.25, P=0.045) than the low SII group in patients with HCC (Figure 5).

### Relationship between preoperative SII and tumor diameter

**Figure 6:**
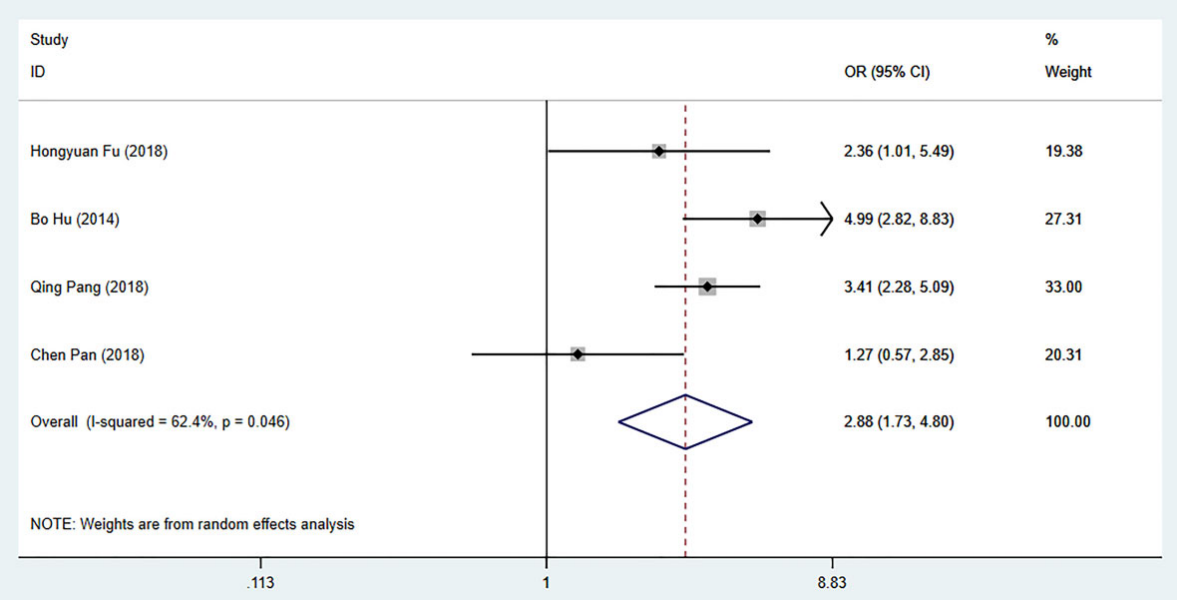
Forest plot of the relationship between preoperative systemic immune-inflammation index and tumor diameter. See Supplementary Table S2 for reference numbers.

Four studies (7,10,12,13) reported the relationship between SII and tumor diameter (5 cm was the cut-off value). Due to the apparent heterogeneity (I^2^=62.4%, P=0.046), a random effect model was used. The results showed that the tumor diameter of the high SII group was larger than that of the low SII group in HCC patients (OR=2.88, 95%CI: 1.73-4.80, P=0.000) (Figure 6).

### Publication bias

**Figure 7:**
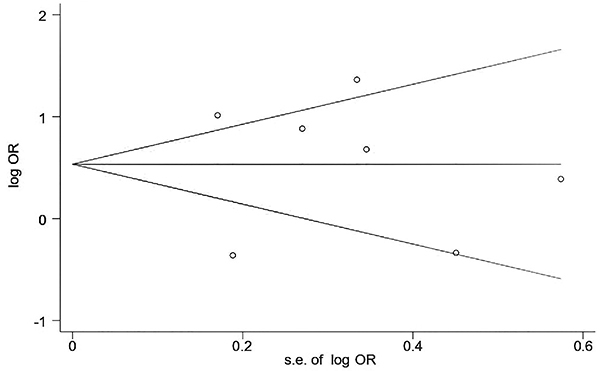
Begg's funnel plot with pseudo 95% confidence limits for publication bias analysis.

Because the number of studies included in this meta-analysis was less than 10, the Begg's test was used to assess publication bias (Figure 7), which indicated that there was no publication bias (P=0.368).

## Discussion

In this meta-analysis, the difference in the selection of preoperative SII cut-off value in the 7 study cohorts was the main reason for heterogeneity. When the two studies with lower cut-off values were excluded, the risk of vascular invasion was greater in the high SII group. On the other hand, when analyzing the two studies with lower cut-off values alone, we found the opposite conclusion. These results suggested that the determination of the cut-off value of SII may directly affect the relationship between preoperative SII and vascular invasion. Therefore, it is necessary to further verify the ability of SII to predict vascular invasion in multicenter studies. It is also of great significance to determine the best cut-off value of SII. An increase of SII means that the number of neutrophils and platelets increases, which leads to the enhancement of tumor cell growth, reproduction, and metastasis. Simultaneously, a decrease in the number of lymphocytes leads to the reduction of the immune system's anti-tumor ability.

There is a close correlation between SII and the prognosis of patients with HCC, but the specific mechanism has not been clarified. It has been reported that neutrophils release cytokines, chemokines, and enzymes, degrade extracellular matrix, reduce cell adhesion, and create conditions for tumor cell invasion. Through physical anchoring, neutrophils promote the adhesion of tumor cells to endothelial cells, resulting in the migration of tumor cells (14). Platelets will form a protective film on the tumor cell surface to avoid the damage of blood flow shear force and the attack of the immune system, and, at the same time, induce the epithelial-mesenchymal transformation of tumor cells to enhance their invasiveness. Platelets can also release pro-angiogenic factors and promote tumor angiogenesis to meet tumor cells' nutritional needs (15). Lymphocytes, as the main cells of the immune response, T cell-mediated cellular immunity, B cell-mediated humoral immunity, and natural killer cells constitute the anti-tumor defense line. A high SII score means that the inflammatory response is enhanced or the immune response is weakened, reflecting the biological invasiveness of the tumor and a higher risk of vascular invasion and tumor growth.

It has been reported that macrovascular invasion and MVI increase postoperative recurrence risk in patients with liver cancer by 15 times and 4.4 times, respectively (16). Macrovascular invasion refers to the tumor thrombus formed in the main branches of the portal vein. The Japanese Hepatocellular Cancer Research Association (17) divides it into four types according to the degree of development of the portal vein tumor thrombus. In the study of Sumie et al. (18), patients with HCC were divided into two groups: non-MVI and mild-MVI groups (1-5 MVI) and severe MVI groups (>5 MVI), and results showed that MVI was negatively correlated with survival and non-recurrence survival. Even if MVI occurs, it does not necessarily affect the patient's prognosis. In Iguchi et al. (19), patients with liver cancer were divided into three groups according to the number of tumor cells suspended in blood vessels: high-MVI group (≥50 tumor cells), low-MVI group (<50 tumor cells), and non-MVI group. The results showed that only high MVI was a risk factor for a worse prognosis of liver transplantation patients. Similarly, Lee et al. (20) found that MVI did not affect tumor recurrence in 38 patients with hepatocellular carcinoma after liver transplantation. Therefore, in the guidelines for standardized pathological diagnosis of primary liver cancer in China (2015 edition) (21), the MVI was redefined as solid nests of ≥50 tumor cells in the vascular lumen lined with endothelial cells under the microscope, and according to the number and distribution of MVI, it is divided into three grades: M0: no MVI; M1 (low risk group) ≤5 MVI and MVI distance from tumor ≤1 cm; and M2 (high risk group) >5 MVI or MVI distance from tumor >1 cm. The study of the dose-effect relationship between preoperative SII level, macrovascular invasion grade level, and MVI grade level, and the determination of the best critical value of SII will further improve the accuracy of preoperative prediction of vascular invasion.

There were some limitations in this study. First of all, the studies included in this meta-analysis were all retrospective studies, with the inevitably limitations and deviations in the original data, which reduces the intensity of the argumentation. Secondly, the HCC patient population in this study was from China, which limits the findings of other countries. Third, at present, most studies determined the critical value of SII based on the prognosis of HCC patients and there is no standard value, so there can be differences in the conclusions of the studies. Finally, as there are few studies on MVI as the outcome index, the outcome still needs to be further verified in a large population sample.

In summary, our results showed that a high SII may be a predictor of vascular invasion in patients with HCC, especially the occurrence of MVI, which is helpful for clinicians to make a reasonable individualized treatment plan. However, considering the limitations of this meta-analysis, it is necessary to explore the quantitative relationship between SII and vascular invasion and determine the best cut-off value in a multicenter prospective study.

## Supplementary Material

Click here to view [pdf].
